# Genetic Modification of T Cells

**DOI:** 10.3390/biomedicines4020009

**Published:** 2016-04-20

**Authors:** Richard A. Morgan, Benjamin Boyerinas

**Affiliations:** Bluebird bio, 150 Second Street, Cambridge, MA 02141, USA; BBoyerinas@bluebirdbio.com

**Keywords:** CAR (chimeric antigen receptor) T cells, immunotherapy, retroviral vector, lentiviral vector, CD19 CAR

## Abstract

Gene transfer technology and its application to human gene therapy greatly expanded in the last decade. One area of investigation that appears particularly promising is the transfer of new genetic material into T cells for the potential treatment of cancer. Herein, we describe several core technologies that now yield high-efficiency gene transfer into primary human T cells. These gene transfer techniques include viral-based gene transfer methods based on modified *Retroviridae* and non-viral methods such as DNA-based transposons and direct transfer of mRNA by electroporation. Where specific examples are cited, we emphasize the transfer of chimeric antigen receptors (CARs) to T cells, which permits engineered T cells to recognize potential tumor antigens.

## 1. Introduction

The field of cancer immunotherapy is in the midst of a renaissance, with both passive and active immunotherapies yielding promising results in clinical trials. Treatment with chimeric antigen receptor-expressing T cells (CARTs) is one such therapeutic intervention that has the potential to permanently alter the face of cancer treatment. While we are just now beginning to understand the complexities involved with implementation of autologous cell transfer therapies such as CART, this type of therapy is dependent on efficient, stable, and safe gene transfer platforms. The major objectives of CART therapy are to achieve tumor eradication in the absence of any severe adverse events and to produce a durable response that can provide continued protection in the event of outgrowth of minimal residual disease. Transfer of a synthetic gene that codes for the chimeric antigen receptor is the first step towards meeting these objectives.

Gene transfer technology has advanced rapidly from simple physical-chemical laboratories methods in the 1970s and 1980s to the sophisticated viral and non-viral methods currently in clinical practice. The ultimate goal of these methods is to achieve high transgene expression in the absence of overtly toxic or oncogenic events. Herein, we review multiple gene transfer methodologies that are being applied in human gene therapy clinical trials transferring chimeric antigen receptors (CARs) into T cells for the treatment of B cell malignancies. The methods described include three viral vector gene transfer technologies (alpha-retrovirus, gamma-retrovirus, and lentivirus), transposons, and mRNA electroporation. We describe the advantages and drawbacks of each system with an emphasis on achieving durable transgene expression in the absence of clinical safety concerns.

## 2. Viral Vectors

Harold Varmus and Michael Bishop’s discovery in 1976 that the oncogenic activity of Rous sarcoma virus (RSV—an alpha retrovirus) is the result of viral-mediated transfer of non-viral DNA provided the foundation for use of retroviral vectors in synthetic biology. Viral vectors of the family *Retroviridae* are now the most commonly used vectors for gene therapy applications, with over 500 registered clinical trials utilizing one of these vector systems in 2014 [1]. The major advantages of viral gene transfer vectors are the relative ease of manufacture and production as well as their capacity to stably integrate genetic material into the host genome. In order to comply with clinical safety standards, viral vector platforms must demonstrate replication incompetence, low genotoxicity, and low immunogenicity.

### 2.1. Gamma Retroviral Vectors

The first gene therapy clinical trials to be considered a success utilized murine leukemia virus (MLV), a gamma retrovirus, as a gene transfer vehicle to treat severe combined immunodeficiency (SCID)-X1 in 11 children [2]. While SCID was successfully corrected in these patients, a significant number developed leukemia due to insertional mutagenesis mediated by the vector in transduced hematopoietic progenitors. These results, at once encouraging and troublesome, underscored a need to develop greater understanding of retroviral integration events in order to manufacture vectors with significantly lower oncogenic potential.

The viral family *Retroviridae* contains seven members with two of these retroviruses, the gamma retrovirus and the lentiviruses (discussed in the next section), being successfully adapted as clinical gene transfer vectors for the treatment of B cell malignancies. All retroviruses are obligate parasites that consist of lipid-enveloped particles comprising a single-stranded diploid RNA genome composed of coding sequences and *cis-*acting regulatory sequences [3]. Two defining characteristics of retroviruses make them particularly suited to act as vectors for gene transfer: (1) most of the viral genome can be replaced with a transgene or transgenes of interest; and (2) upon transduction, the viral genome is permanently integrated into the host cell genome. For these reasons, simple gamma retroviruses such as the Moloney murine leukemia virus (Mo-MLV) were among the first to be successfully engineered to serve as advanced packaging systems for gene transfer [4].

**Figure 1:**
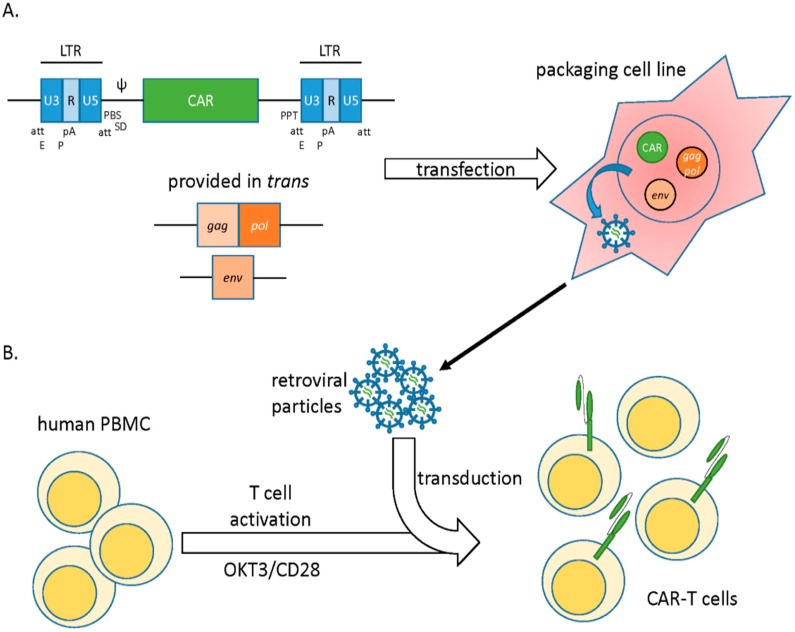
Gamma retroviral vectors. (**A**) Genomic structure of MLV-derived γ-retroviral vectors. Essential genes *gag*, *pol*, and *env* are removed from the viral backbone and provided in *trans* for viral production. Transgene encoding CAR is introduced in place of the viral genes*.* A packaging cell line is transfected with the vector carrying the CAR transgene, packaging and *env* helper plasmids. If desired, selective antibiotic pressure is utilized to select for plasmid integration and generate stable virus-producing lines for large-scale production. (**B**) Retroviral particles are collected from the cell culture supernatant and used to transduce stimulated T cells (OKT3/CD28 blasts). After genomic integration, the CAR is stably expressed on the surface of T cells. att, integration signal; E, enhancer; P, promoter; pA, polyadenylation signal; PBS, tRNA primer-binding site; SD, Splice donor; Ψ, encapsidation signal; PPT, polypurine tract.

To generate a vector, the gamma retroviral coding sequences are replaced by a transgene of interest. Successful packaging of the recombinant viral genome into particles requires the concomitant expression of viral genes *gag*, *pol*, and *env*, which encode for capsid proteins, replication enzymes, and envelope glycoproteins, respectively. These are provided in *trans* as heterologous subgenomic helper plasmids devoid of any packaging signal. Separation of coding sequences and the regulatory sequences into distinct nucleic acid molecules limits their remobilization into replication competent retroviruses (RCRs), thereby increasing safety [3,5]. When transfected into a packaging cell line, vector plasmids allow for synthesis of many copies of the viral genome, which are subsequently packaged into viral particles by the structural proteins (Figure 1).

The tropism of the viral particles, *i.e.*, the ability to preferentially transduce one cell type over another, is dictated by the *env* gene. The process of pseudotyping allows for the substitution of one envelope for another from a different retrovirus species, thereby conferring a broad host range or tropism to a given vector. For example, substituting the murine amphotropic MLV envelope glycoprotein with that from the gibbon-ape leukemia virus (GALV) or the endogenous feline retrovirus RD114 allows for more efficient transduction of human cells of the hematopoietic lineage. The events following transduction closely resemble those of a true infection. Upon the fusion of viral and host membrane, the virion core is released into the cytosol and transported along the microtubules to reach the nucleus [6]. A disrupted nuclear membrane is absolutely crucial for its entry into the nucleus, and as such productive transduction by gamma-retroviral vectors is strictly dependent on target cell mitosis [3].

Gamma retroviral vectors have been efficiently used to express chimeric antigen receptors (CARs) in T lymphocytes. Typically, peripheral blood mononuclear cells (PBMCs) from patients are stimulated with anti-CD3 and anti-CD28 monoclonal antibodies (MAb) along with exogenous IL-2 to select and expand T cells within peripheral blood lymphocytes (PBLs) [7]. These are subsequently transduced with vector supernatant generated from high-titer vector packaging cell lines (VCP) such as PG13. Such packaging cell lines are generated via transient transfection of the CAR construct in combination with essential viral genes (*gag*, *pol*, *env*) that are provided in *trans*. If desired, stable integration of these transgenes is selected for via the use of standard antibiotic resistance cassettes. When combined with inducible promotor systems, this approach allows for the generation of stable producer cell lines where virus production is induced via addition of a small molecule such as tetracycline. This method permits the generation of large numbers of T cells that express high levels of the CAR (Figure 1) [8,9]. Permanent and long-lived CAR expression can be boosted by retroviral enhancer/promoter elements in the long terminal repeats (LTRs) or the incorporation of the woodchuck hepatitis virus post-translational regulatory element (WPRE), among others [7,10]. Immune responses to retroviral proteins can be diminished by the use of a “clean vector backbone” that is free from residual coding sequences, a method that also lessens recombinogenic sequence overlap [11]. The anti-tumor efficacy of gamma retroviral vector generated CART cells are currently being tested in numerous clinical trials (e.g., see NCT01087294, NCT01454596, and NCT01822652 at www.clinicaltrials.gov).

Gamma retroviral vectors are commonly used in gene therapy applications due to their ability to achieve high rates of transduction and significant transgene expression that persists over time. The safety of gamma retroviral vectors is also being carefully monitored, especially in light of adverse events that were associated with insertional mutagenesis in trials targeting hematopoietic stem cells for human severe immunodeficiency (SCID)-X1 [12]. Due to their intrinsic ability to integrate close to cellular gene promoters, gamma retroviral vectors carry an innate ability to perturb the genomic region flanking the integration site, which can result in neoplastic transformation should integration occur in or near a proto-oncogene [13]. Genome-wide analysis studies have demonstrated that gamma retroviruses preferentially integrate near transcription start sites and CpG islands, and that this preferential integration profile increases the chances of oncogenic transformation compared to a more random integration profile [14]. This potential for oncogene activation can be caused by several mechanisms, including promoter insertion, promoter activation, and gene transcript truncation.

**Figure 2:**
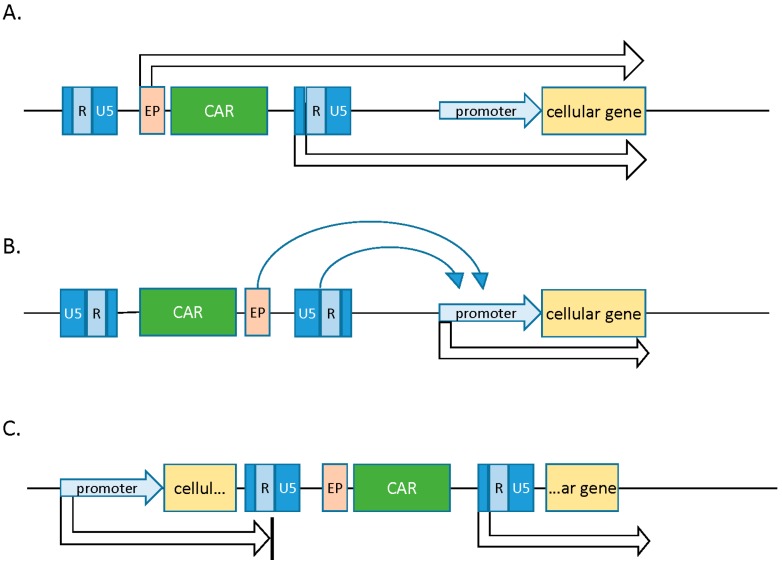
Modes of insertional mutagenesis. (**A**) Promoter insertion—expression of a cellular gene is upregulated when an insertion is upstream and in frame with the cellular ORF (open reading frame). Read-through from either the endogenous promoter or the viral LTR can induce aberrant gene expression. (**B**) Promoter activation—activity of a cellular promoter is influenced by enhancer elements in the viral LTR. This effect is not dependent on orientation or frame agreement and can function at a distance of several kilobases; (**C**) intronic insertions can lead to the production of truncated cellular transcripts. Adapted from Suerth *et al.* [1].

In the case of promoter insertion, genotoxicity results from insertion of strong viral promoter units directly upstream of target cellular transcription units. Oncogenic activation in this scenario is restricted to insertion events upstream of and in frame with the resultant oncogene, as the viral promoter directly influences transcription of a host gene. Promoter activation, on the other hand, is the result of the enhancer in the viral LTR acting on the promoter of a proto-oncogene. This effect is not dependent on orientation or frame agreement between the insertion and target promoter, and it can function at a distance of several kilobases. Promoter activation has been implicated as the most common mutational event observed in gene therapy clinical trials. Lastly, insertions into intronic regions can lead to aberrant splicing events and truncated transcripts missing important regulatory elements (Figure 2). Loss of transcript in the 5′ or 3′ UTRs (untranslated regions), for instance, can lead to abrogation of microRNA regulation and resultant oncogenesis [1].

One strategy to increase the safety profile of gamma retroviral vectors is to alter the integration profile itself. Until recently, the molecular mechanisms driving gamma retrovirus integration profiles were not well elucidated. It was recently demonstrated, however, that the gamma retroviral integrase interacts with human BET proteins to mediate genomic integration. BET proteins bind to acetylated H3 and H4 tails, which are highly enriched at transcriptional start sites, and tether the MLV integrase to these sites. Treating cells with JQ-1, a drug that interrupts BET binding to modified histones, was shown to reduce MLV integration frequencies at transcriptional start sites [15]. Additional studies have demonstrated that truncation of the MLV integrase so that it no longer associates with BET proteins functions to alter the MLV integration profile and significantly reduces the preference for transcriptional start sites near oncogenes [16,17]. While these studies are pre-clinical in nature, they suggest the feasibility of redistributing gamma retroviral integrations in order to reduce potential oncogenicity. Further investigation is warranted to determine whether these types of modified gamma retroviral vectors will provide the same level of clinical efficacy as previously demonstrated with non-modified vectors.

The types of insertional mutagenesis described above have been observed in gene therapy trials attempting to modify hematopoietic stem cells. Mature lymphocytes, however, are known to resist transformation events owing to pro-apoptotic and epigenetic mechanisms that prevent clonal outgrowth [18]. Indeed, there was no evidence of vector-induced immortalization of cells or enrichment for specific integration sites in a long-term study using gamma retroviral-engineered T cells for HIV [19]. Nevertheless, novel strategies to further improve safety including the use of insulator sequences that hinder viral promoter activity and self-inactivating (SIN) retroviral vectors are currently being tested in pre-clinical studies [20,21]. These types of safety modifications will be discussed in more detail below as they also pertain to lentiviral vector systems.

### 2.2. Lentiviral Vectors

**Figure 3:**
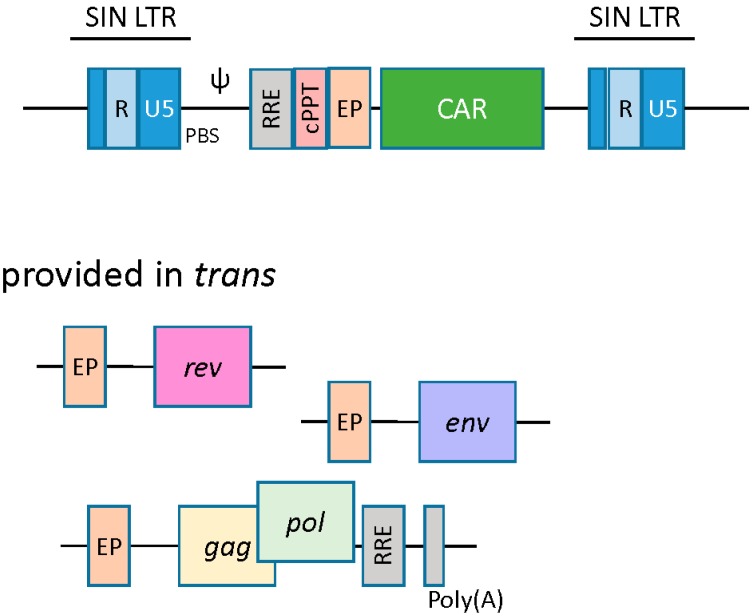
Lentiviral vectors. Lentivirus based vectors are similar to their retroviral counterparts. A split packaging system is utilized with a packaging cell line to produce viral particles. An accessory plasmid provided in *trans* unique to lentivirus is *rev*, which enhances nuclear export of gag-pol transcripts. Another component unique to lentiviral vectors is the central polypurine tract (cPPT), which facilitates nuclear import of the preintegration complex. EP, eukaryotic promoter; RRE, rev response element; cPPT, central polypurine tract; PBS, tRNA primer-binding site; Ψ, encapsidation signal.

Lentivirus-based vectors are structurally similar to their gamma retroviral counterparts, wherein the essential viral genes are replaced with a transgene of interest, and the viral genome is stably integrated into the host cell. In addition to *gag*, *pol*, and *env*, lentivirus-based vectors require the *trans* presentation of the *rev* gene. Rev protein binds to the rev responsive element (RRE) and enhances nuclear export and expression of gag-pol transcripts [22]. Another *cis-*acting element unique to the lentiviral vector is the central polypurine tract (cPPT)/central termination signal (CTS), the function of which is to facilitate nuclear import of the preintegration complex upon infection (Figure 3) [23].

As opposed to gamma-retroviruses, lentiviral vector transduction is therefore not governed by cell division, allowing effective transduction of a wide range of cell types, including non-cycling terminally differentiated cells. Pseudotyping with vesicular stomatitis virus g-protein (VSV-G) further broadens vector tropism, as the cellular receptors for VSV-G are ubiquitously expressed on human cells [24]. Of note, it has been shown, somewhat counterintuitively, that VSV-G pseudotyped vectors do no transduce T cells or HSCs (hematopoietic stem cells) with high efficiency unless the target cells are activated and induced to proliferate. This mystery was, at least partially, solved when LDL-R (low density lipoprotein receptor) was identified as the human cellular receptor for VSV-G, and it was shown that LDL-R expression is relatively low on resting HSCs and T cells. Expression of LDL-R is upregulated in cells that have been activated to proliferate, making them more susceptible to transduction by VSV-G pseudotyped vectors [25].

The advent of lineage-specific promoters or cell-type-specific lentiviral vectors may improve the specificity, efficacy, and safety of lentiviral vectors [25,26,27]. To specifically target T cells, for example, an anti-CD3scFv antibody fragment was fused to an MLV glycoprotein. Interaction of the anti-CD3 with the T cell receptor (TCR) complex proteins induced signaling in the resting T cells and allowed 100-fold more gene transfer than unmodified vectors [28]. Improved pseudotyping may affect transduction efficiencies. Lentiviral vectors pseudotyped with Edmonston measles virus (MV) glycoprotein H and F, for example, outperformed VSV-G pseudotyped vectors for the transduction of IL-7 prestimulated and quiescent T cells [22,29].

The most commonly used lentiviral vectors are based on the human immunodeficiency virus (HIV). Naturally, concerns over the generation of replication competent particles have necessitated the development of increasingly sophisticated lentiviral vectors with multiple safeguards. Similar to the gamma retroviral vectors, split-component systems that involve the separation of essential viral genes from the *cis*-regulatory sequences have reduced the chances of recombination and subsequent remobilization of viral particles. Nonetheless, the FDA (Food and Drug Administration) requires that all therapeutic products created via lentiviral transduction be thoroughly tested for the presence of replication competent virus. Most recently, hybrid lentiviral vectors derived from non-human lentiviruses (simian, equine, feline, caprine, and bovine) are being considered as potentially safer alternatives as their parental viruses are apathogenic in humans, but when optimized can efficiently transduce human cells [30].

The production of CAR-engineered T cells by lentiviral vectors is similar to that of the gamma retroviral vectors in certain respects. A conditional packaging construct expressing *gag-pol* is transfected with vector plasmid carrying the CAR transgene along with *rev* and *env* packaging constructs (Figure 3) [31,32]. Downstream vector supernatant processing may require ultracentrifugation, anion exchange chromatography, and gel filtration to produce a high-titer lentiviral supernatant [22]. Resting T cells are refractory to lentivirus transduction and need to be stimulated to enter the G_1b_ phase of the cell cycle. Again, anti-CD3 and anti-CD28 antibodies can be used to coax T cells out of their G_o_ status and render them susceptible to lentiviral transduction [33]. Productive transduction and genome integration allows for stable expression of CAR transgene in T cells [34]. Lentiviral generated CART cells targeting CD19 antigen have shown remarkable anti-tumor efficacy in clinical trials [35,36].

The lentiviral vector genomic integration site preference differs from gamma-retroviral vectors and does not show a preference for gene promoter regions. While the risk of insertional mutagenesis is lower with lentiviral vectors [37], SIN vectors with disrupted LTRs further reduce this probability [37]. In these systems, the enhancer capacity of the LTR is greatly diminished via removal of a large portion of the 3′ U3 region containing viral transcriptional enhancers [2]. The LTR thus does not act as a promoter, and expression of the transgene is driven by addition of an internal promoter such as CMV or MND.

Recently, Cesana *et al.* investigated the genotoxicity and relative immunogenicity of SIN lentiviral vectors in a tumor-prone mouse model [38]. Using systemic delivery in Cdkn2a knockout mice, they demonstrated that SIN vectors induced significantly fewer oncogenic events driven by enhancer-mediated oncogene activation as compared to vectors with wild-type LTR regions. They also showed that the rate of oncogenic events associated with SIN vectors correlated with the strength of internal promoter used, suggesting that SIN vectors can still induce oncogene activation depending on internal promoter design. This activation could be further reduced by including synthetic chromatin insulators within the SIN LV LTRs. These insulators function to attenuate read-through from internal promoters, and significantly reduced the occurrence of oncogene activation events resulting from SIN LV integrations. Even insulated SIN lentiviral vectors, however, were demonstrated to induce oncogenesis through various processes resulting in tumor suppressor inactivation [38]. These results demonstrate the feasibility of producing lentiviral vectors with lower genotoxicity, but show that greater levels of control over integration are necessary to completely eliminate the possibility of oncogenic transformation.

One strategy to circumvent genotoxicity associated with lentiviral vectors is to completely eliminate integration capacity. In this scenario, the integrase is either mutated or eliminated, resulting in a viral construct that can transiently express a transgene of interest episomally. The current drawbacks to these vector systems are lower overall transgene expression and loss of expression in rapidly dividing target cells. Low transgene expression can be compensated for by the inclusion of stronger promoters. This has proven difficult to optimize, however, as these vectors can still illegitimately integrate into the host genome (albeit at significantly lower frequency), where strong promoters can contribute to oncogenic events [39]. When transient expression of the transgene is preferred, however, integration-deficient vectors can be particularly effective [29].

Recent studies have investigated whether certain vector and/or procedural modifications can increase the durability of transgene expression from integration-deficient lentiviral vectors. One particular modification involves the addition of scaffold/matrix associated regions (S/MARs) from the human β-interferon locus to a non-integrating lentiviral vector. S/MAR elements can function to recruit host cellular factors to viral episomes to promote replication and mitotic stability. Verghese *et al.* demonstrated that non-integrating lentiviral vectors containing these elements can generate significant transgene expression that persists clonally for more than 100 cellular divisions *in vivo* [40]. Transgene expression is not stable in a significant proportion of transduced cells and does eventually decrease as compared to control integrating vectors, but these data suggest that modifications aimed at increasing episome stability in dividing cells are feasible and warrant further investigations.

Non-integrating lentiviral vectors have also shown promise to drive expression of technologies such as site-directed integration systems (zinc-finger nuclease, TALEN). Multiple groups, for instance, have demonstrated the use of integration-deficient lentiviral vectors to deliver zinc-finger nucleases (ZFN) and template DNA to induce ZFN-mediated targeted integration events [41,42]. These studies demonstrated targeted transgene integration into the host genome, as well as an advantage in viability as compared to current electroporation methods frequently used to introduce plasmid-based ZFNs. In transformed cell types such as K562, high transgene expression from non-integrated episomes is sufficient to drive significant ZFN-mediated integration. The drawback with these systems at present is relatively low efficiency of transgene integration in primary cells of the hematopoietic lineage. This was demonstrated to be the result of significantly less ZFN transcript per vector copy number in these primary cell types, suggesting that optimization of expression will likely increase integration efficiency. Utilizing stronger internal promoters or combining this technique with the S/MAR modifications described above, for instance, might allow for ZFN- or TALEN-mediated modification of a significant percentage of T cells in the absence of genotoxicity.

### 2.3. Alpha Retroviral Vectors

While gamma retrovirus and lentivirus are the most widely used viral vector platforms for genetic modification of T cells, other viral platforms have been developed to introduce genetic material into target cells. Once such viral vector platform in pre-clinical development utilizes alpha retrovirus, a genus of the *Retroviridae* family that includes the aforementioned Rous sarcoma virus. An alpha retrovirus-based gene delivery system holds several potential advantages over other retroviral-based vectors. First and foremost, alpha retroviruses display a more random and neutral integration pattern as compared to other retroviruses, with no detectable enrichment in genes or transcriptional start sites [1]. Suerth and colleagues have developed a self-inactivating alpha retrovirus gene delivery platform, based on the avian leukosis virus and pseudotyped with VSV-G, that lacks replication competency in mammalian cells [43]. They have demonstrated a neutral integration profile in murine HSCs, durable transgene expression, and significantly reduced oncogenic transformation (in a transformation prone mouse model) as compared to gamma retroviral and lentiviral vectors [44]. Furthermore, this vector system is devoid of viral splice elements, and the split packaging system contains significantly reduced sequence overlap amongst viral and packaging plasmids (as compared to other retroviral vector systems), greatly reducing the potential for development of replication competent virus.

A final advantage of alpha retroviral vectors, particularly as compared to lentiviral vectors, is the capability to manufacture stable producer cell lines. The use of producer cell lines significantly decreases chances of recombination events as compared to transient virus production, and it also provides the economic advantages of consistent and robust large-scale virus production. Establishment of similar systems with lentiviral vectors has proved challenging due to long-term silencing of structural lentiviral components in producer cell lines [1]. The advantages of alpha retroviral vectors have yet to be tested in a clinical setting, but they appear to be an intriguing and potentially safer alternative to currently utilized retroviral gene therapy vectors.

## 3. Non-Viral Vector Systems

The viral gene transfer systems described above possess many advantages that have made them the vectors of choice for genetic modification of T cells. Their pathogenic ancestry and potential for insertional mutagenesis, however, poses significant regulatory hurdles for implementation in human clinical trials. It is for this reason that significant pre-clinical and clinical research has been devoted to developing alternative vector systems that are not dependent on viral architecture.

### 3.1. Transposons

**Figure 4:**
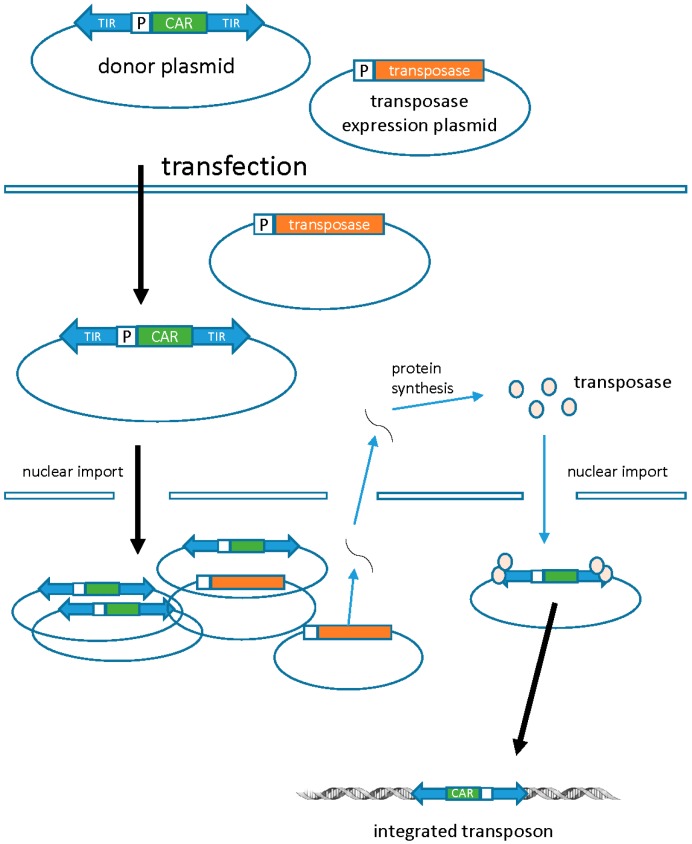
Transposons. Transposons are dual component systems composed of one plasmid carrying the CAR (transposon) and the other carrying the transposase. The transposase acts on the terminal inverted repeats flanking the CAR, which leads to excision and subsequent integration at a TA dinucleotide sequence in the target cell genome. DNA plasmids carrying the CAR (transposon) and transposase are electroporated into PBMCs. Following transposition and stable genomic integration, the CAR protein is expressed on the surface of the T cell. TIR, terminal inverted repeats; P, promoter. Adapted from Cai & Mikkelsen [54].

DNA transposons are a non-viral alternative for stable gene transfer. In nature, transposons are discrete pieces of DNA containing the transposase gene flanked by terminal inverted repeats (TIRs) that contain the transposase binding sites. Transposase binds to the TIRs and “cuts” the transposon from one location and “pastes” it into a new site (known as transposition). Transposon-based vector systems like the *Sleeping Beauty* (SB) system are built to utilize transposition as a way to introduce a transgene of interest into the host genome. This is achieved by introducing the transgene between the transposon TIRs, which are subsequently mobilized by supplementing transposase in *trans* as an expression plasmid, mRNA, or protein. SB transposons are integrated into the chromosomal DNA in an intact form, eliminating the risk of rearrangements or random mutations that are inherent to the reverse transcription of retroviral vectors (Figure 4) [45]. Similar to the retroviral vectors, stable genomic integration can lead to long-term and efficient transgene expression. Genomic integration of the SB transposons is fairly random and additional strategies may be required to improve the safety of these non-viral vectors.

Safety-enhancing strategies include the use of modified transposases that can mediate site-specific targeting of the transgene into a “safe haven” to limit genotoxicity or, alternatively, allow transient expression of transposase to lower the risk of “rehopping” [46]. Other options include using transcriptional regulators to prevent activation/disruption of genes flanking the insertional site. The SB system may be a safer alternative to viral vectors owing to its non-pathogenic origin, inherently low enhancer/promoter activity, and minimal epigenetic modifications at the insertion site [45]. In addition, readily available clinical reagents and low manufacturing costs have launched the SB system as a strong contender against viral vectors that are often limited by a lengthy manufacturing time, high cost, and other regulatory issues.

T cells have been successfully modified by the SB transposon to express CD19-specific CAR. CD19-CAR (transposon) and transposase plasmid DNA were electroporated into peripheral blood mononuclear cells (PBMCs), which were subsequently propagated on γ-irradiated CD19+ artificial antigen-presenting cells (aAPCs). This allowed for the generation of clinically sufficient numbers of CART cells within 3–4 weeks after electroporation [47]. The safety and efficacy of these T cells are currently being tested in patients with advanced B-lineage lymphoid malignancies post autologous hematopoietic stem cell transplantation [48].

The *piggy-bac* (PB) transposon system is an alternative to the SB system for gene delivery into mammalian cells. Compared to the SB system, the PB system showed a larger cargo capacity and more efficient transposition activity in preclinical studies. CART cells specific for CD19 and HER2 have been successfully generated using the PB system [49,50]. Parallel comparisons are needed to determine the superiority and, more importantly, the safety of PB-system-derived CART cells over those from the SB system, especially in light of the fact that PB transposons tend to integrate closer to transcription start sites [51].

A third transposase platform developed to deliver genetic material to T cells is *Tol2.* As opposed to SB and PB transposases, which integrate preferentially at TA and TTAA sites, respectively, *Tol2* transposase demonstrates a random integration profile. This system, as well as the PB system, is also not prone to the overproduction inhibition that can occur in the presence of excess amounts of SB transposase. Furthermore, *Tol2* transposons can carry a comparable cargo load (up to 100 kb) to PB transposons, an advantage over the SB system, where cargo load limits are smaller. The *Tol2* system has been used to produce human CD19-directed CART cells that function well in mouse models, but the system has not yet been optimized to produce the number of cells needed for application in the clinic [52].

The current limitation of transposon-mediated gene transfer is reliance on electroporation for nucleic acid delivery and the resultant low frequency of cells carrying integrations. This drawback is partially mitigated by expanding transduced CART cells on aAPCs, but the excess manufacturing time this requires is not ideal, and the prolonged exposure to antigens during the production process may produce cells with a more differentiated effector phenotype that reduces long-term memory function. One possible way to overcome this limitation is to use non-integrating lentiviral vectors to deliver both the transposon and the transposase. This type of system has been successfully implemented in primary human cells to drive transposition using the PB system [53]. Of note, when this system was compared to electroporation delivery, it was demonstrated that transfected cell populations contained cells with multiple integration events, while virally transduced populations contained cells with only a single integration event. This was likely due to the much lower level of transposase expression achieved with the viral delivery system [53,54]. While the efficiency of this system is still significantly lower than gene delivery using an integrating viral vector, the ability to limit integration events to one per cell will reduce the potential for genotoxicity in target cells.

### 3.2. mRNA Electroporation

Messenger RNA (mRNA) transfer-mediated gene expression has been gaining popularity as a safer alternative to viral and plasmid DNA (pDNA)-based approaches. mRNA transfer represents a cytoplasmic expression system, *i.e.*, it does not need to enter the nucleus to mediate its function. The advantages of this system therefore include: (1) the ability to transfect quiescent or slow-proliferating cells; (2) no genomic integration and therefore a very low probability of insertional mutagenesis; and (3) a transient expression profile that diminishes over time. Finally, the relative ease of mRNA engineering has made this system particularly attractive in the context of gene/cell therapy [55].

The limiting feature of an mRNA transfer strategy is the unstable and relatively short-lived nature of the mRNA molecule itself, which can lead to a rather transient expression of the encoded protein. This may, however, be advantageous in the context of CART cell therapy in certain contexts. Constitutive expression of some CARs (as with viral vectors) has resulted in serious adverse effects due to the unintended cross-reactivity of the CAR molecule with normal tissues [56]. Transient CAR expression could therefore improve safety especially when the tissue cross-reactivity of a particular CAR is not known. This strategy could allow for a quicker regulatory path for newly designed CAR molecules, and one could envision Phase I clinical trials utilizing transient CAR expression to assess the potential off-tumor reactivity of new CART drug products. Lack of toxicity in these trials could be used to “greenlight” an antigen target for more durable CAR therapies. The non-viral nature of mRNA electroporation may have a less cumbersome regulatory approval process for Phase I trial development.

*In vitro* transcribed (IVT) mRNA can be synthesized with modifications that increase its stability and allow for longer periods of expression. Transfer can be mediated by either disruption of the cell membrane (electroporation, gene gun) or by endocytosis (cationic carriers) [55]. Electroporation of mRNA has been successfully adapted in various cell types, including pre-stimulated T-cells. In a preclinical model, mesothelin mRNA CAR-electroporated autologous patient T cells mediated regression of disseminated mesothelioma xenografts [57]. In another study, HER2/*neu* mRNA CAR-electroporated T cells mediated superior anti-tumor effects in a breast xenograft model compared to HER2/*neu* antibodies [58].

A single injection of CD19 mRNA CAR-electroporated T cells showed comparable initial efficacy to lentivirus-transduced CARTs in a xenograft model of aggressive leukemia [59]. CART cells were only detectable in these mice for one week on average, demonstrating the transient nature of mRNA modification. The increased safety profile of transient mRNA expression also allows for multiple frequent injections of transient CART cells. It has recently been demonstrated that an initial loading dose of transient CD19-targeted CART cells, followed by weekly maintenance doses, extends therapeutic efficacy significantly as compared to a single infusion [60]. Therapeutic efficacy of stably transduced CD19 CART cells, however, was superior to the transiently expressing CAR cells in these experiments, likely due to their significant expansion *in vivo* upon antigen encounter. These data support the use of mRNA expression systems for initial validation of novel CARs, but suggest that responses will likely lack durability. The efficacy of mRNA electroporated CART cells is currently being tested in Phase I clinical trial (see NCT01897415 at www.clinicaltrials.gov).

## 4. Summary

Each of the T cell gene engineering techniques described here has its advantages and disadvantages. The gamma-retoviral vectors were the first gene transfer system used in humans (in the context of tumor-infiltrating T cells) and have a >25-year safety record in human T cells, with no adverse events being associated with the gene transfer system itself. The lentiviral vectors have a theoretical safety advantage in comparison to gamma retroviruses and can transduce less stimulated (*i.e.*, less differentiated) T cells. The alpha-retrovirus has yet to be used in CAR manufacture, but has the potential to be the least genotoxic of the viral vectors due to its neutral integration profile. Transposons may eventually be developed into a versatile T cell gene transfer system, but current DNA electroporation methods generally yield poor cell viabilities, requiring extended T cell culture for the generation of large numbers of T cells for potential clinical applications. mRNA electroporation may be the easiest and safest way to introduce CARs into T cells, but its transient nature requires multiple treatment cycles. Given the rapid progress of the field it is certain that the T cell gene transfer methods described here will continue to advance and lead to more effective treatments for cancer.
